# Novel Superhard Boron Nitrides, B_2_N_3_ and B_3_N_3_: Crystal Chemistry and First-Principles Studies

**DOI:** 10.3390/molecules29174052

**Published:** 2024-08-27

**Authors:** Samir F. Matar, Vladimir L. Solozhenko

**Affiliations:** 1 Lebanese German University (LGU), Jounieh P.O. Box 206, Lebanon; 2 LSPM–CNRS, Université Sorbonne Paris Nord, 93430 Villetaneuse, France

**Keywords:** boron nitride, DFT, crystal structure, elastic constants, hardness, phonons, electronic band structures

## Abstract

Tetragonal and hexagonal hybrid sp^3^/sp^2^ carbon allotropes C_5_ were proposed based on crystal chemistry and subsequently used as template structures to identify new binary phases of the B–N system, specifically tetragonal and hexagonal boron nitrides, B_2_N_3_ and B_3_N_3_. The ground structures and energy-dependent quantities of the new phases were computed within the framework of quantum density functional theory (DFT). All four new boron nitrides were found to be cohesive and mechanically (elastic constants) stable. Vickers hardness (*H*_V_), evaluated by various models, qualified all new phases as superhard (*H*_V_ > 40 GPa). Dynamically, all new boron nitrides were found to be stable from positive phonon frequencies. The electronic band structures revealed mainly conductive behavior due to the presence of π electrons of sp^2^-like hybrid atoms.

## 1. Introduction

Carbon allotropes that exhibit mechanical properties similar to those of diamond, in particular, extreme hardness, continue to be a subject of significant interest within the scientific community. Both the predominant cubic diamond and the less common hexagonal form (“lonsdaleite”) exhibit ultrahigh hardness due to the three-dimensional arrangement of *C4* tetrahedra with the pure sp^3^ hybridization of the carbon atoms. In terms of topology, cubic and hexagonal diamonds are the aristotypes, designated as **dia** and **lon**, respectively [1]. This nomenclature is also applicable to other families of carbon allotropes. The introduction of additional carbon atoms results in alterations to the C(sp^3^) lattice of diamond, giving rise to novel C(sp^3^)/C(sp^2^) hybrid allotropes that retain the original physical properties, including the electronic ones, which can lead to induced metallicity [2]. In the case of the nearest neighbors of carbon, boron and nitrogen, equiatomic cubic boron nitride (cBN) has been synthesized, which is half as hard as diamond but exhibits much higher thermal and chemical stability [3].

Research efforts to identify new superhard phases of compounds of light elements require the use of structure prediction programs such as USPEX [4] and CALYPSO [5]. However, novel structures can also be identified through the application of crystal engineering rationale, as presented here. In all cases, such predictions must be validated by the quantitative study of energies and the derived physical properties using first-principles calculations. Over the years, the well-established quantum mechanics framework of density functional theory (DFT) [6,7] has been proven to be the most efficient.

The present paper further develops the field of boron nitrides by proposing novel cohesive and stable hybrid B–N phases. First, tetragonal and hexagonal sp^3^/sp^2^ hybrid C_5_ allotropes were designed by crystal chemistry engineering and subsequently used as templates for the selective substitution of carbon with boron and nitrogen, leading to the sesquinitride B_2_N_3_, which was then transformed into the equiatomic B_3_N_3_ through the insertion of an additional boron atom into B_2_N_3_.

Considering that boron nitride is equiatomic, our challenging prediction of boron sesquinitride is supported to some extent by the previously reported nitrogen-excess tetragonal B_2_N_3_ [8], but no structural details were provided for this compound. The boron nitride B_116_N_124_ fullerene should also be mentioned as a nitrogen-rich B–N phase [9]. Among the boron-rich compounds of the B–N system, it is worth mentioning rhombohedral boron subnitride B_13_N_2_ [10], which is a superhard phase [11].

In the present work, we show that all predicted novel B–N phases are cohesive, mechanically and dynamically stable, and characterized by high Vickers hardness and metallic-like behavior.

## 2. Computational Methodology

The determination of the ground state structures corresponding to the energy minima and the prediction of their mechanical and dynamical properties were carried out within the widely accepted framework of DFT. DFT was initially proposed in two publications: in 1964, Hohenberg and Kohn developed the theoretical framework [6], and in 1965, Kohn and Sham established the Kohn–Sham equations for a practical solution of the wave equation [7].

Based on DFT, calculations were performed within the Vienna Ab initio Simulation Package (VASP) code [12,13] and the Projector Augmented Wave (PAW) method [13,14] for atomic potentials. DFT exchange correlation (XC) effects were considered using the generalized gradient approximation (GGA) [15]. Preliminary calculations with the native DFT-XC local density approximation (LDA) [16] resulted in underestimated lattice constants at ambient pressure and were therefore abandoned. Relaxation of the atoms to the ground-state structures was performed with the conjugate gradient algorithm according to Press et al. [17]. The Blöchl tetrahedron method [18] with corrections according to the Methfessel and Paxton scheme [19] was used for geometry optimization and energy calculations, respectively. Brillouin-zone (BZ) integrals were approximated by a special **k**-point sampling according to Monkhorst and Pack [20]. Structural parameters were optimized until atomic forces were below 0.02 eV/Å and all stress components were <0.003 eV/Å^3^. The calculations were converged at an energy cutoff of 400 eV for the plane-wave basis set in terms of the **k**-point integration in the reciprocal space from *k_x_*(6) × *k_y_*(6) × *k_z_*(6) up to *k_x_*(12) × *k_y_*(12) × *k_z_*(12) to obtain a final convergence and relaxation to zero strain for the original stoichiometries presented in this work, after a systematic upgrade of the structure input file throughout successive cycles of calculations. In the post-processing of the ground state electronic structures, the charge density projections were operated on the lattice sites.

The investigation of the mechanical properties was based on calculations of the elastic properties determined by performing finite distortions of the lattice and deriving the elastic constants from the strain–stress relationship. The treatment of the results was conducted using the ELATE online tool, which is devoted to the analysis of elastic tensors [21]. The program provides the bulk (*B*), shear (*G*), and Young’s (*E*) moduli along different averaging methods; the Voigt method [22] was used here. Two empirical models, Mazhnik–Oganov [23] and Chen–Niu [24], were used to estimate the Vickers hardness (*H*_V_) from the elastic constants.

Vickers hardness was also evaluated in the framework of the thermodynamic model [25,26], which is based on the thermodynamic properties and crystal structure, and using the Lyakhov–Oganov approach [27], which considers the topology of the crystal structure, the strength of covalent bonds, the degree of ionicity, and directionality. Fracture toughness (K_Ic_) was estimated using the Mazhnik–Oganov model [23].

The dynamic stabilities were confirmed by the positive phonon magnitudes. The corresponding phonon band structures were obtained from the high resolution of the tetragonal and hexagonal Brillouin zones according to Togo et al. [28]. The electronic band structures were obtained using the all-electron DFT-based ASW method [29] and the GGA XC functional [15]. The Visualization for Electronic and Structural Analysis (VESTA) program [30] was used to visualize the crystal structures and charge densities.

## 3. Crystal Chemistry

### 3.1. Tetragonal and Hexagonal Pentacarbon Allotropes

**Figure 1:**
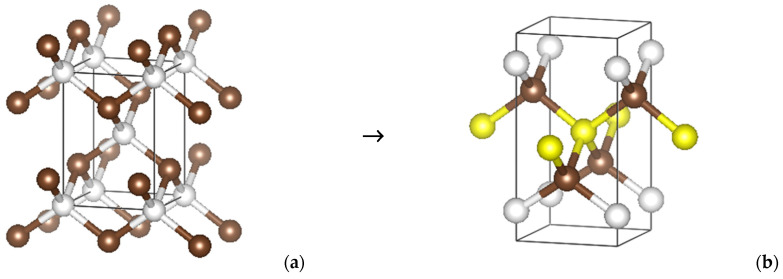
Schematic transformation of *tet*-C_4_ (**a**) to *tet*-C_5_ (**b**) (see Table 1 and text for details). The additional yellow spheres result from the decrease in symmetry.

***tet*-C_5_**. Recently, the body-centered tetragonal carbon allotrope C_4_ (*tet*-C_4_) in the space group *I*-4*m*2 (No. 119) [31] (Figure 1a) has been proposed to serve as a template for the design of the related phases. The four atoms of the unit cell are detailed in Table 1, considering the structure in simple tetragonal arrangement. The subsequent transformation into C_5_ consists of keeping the body center carbon (C1 at ½, ½, ½), which becomes C(1c) in the Wyckoff position (following the transformation arrows), as well as the two C2s, whereby C2a and C2b become C3(2g), and the z coordinate becomes ±z′ instead of ±¼. The additional carbon C2 is provided by parameterizing the z position of C1b, resulting in a two-fold C2(2e) at 0, 0, ±z. After full geometry relaxation, the resulting structure retains the tetragonal symmetry now resolved in space group *P*-4*m*2 (No. 115). The structure is shown in Figure 1b, with colored spheres corresponding to the three carbon sites given by the coordinates in Table 1, i.e., with well-determined z = ±0.854 and zʹ = ±0.314. Using the TopCryst crystallography package [32], *tet*-C_5_ was identified with **3,4^2T1-CA** topology. A similar topology was found for another tetragonal carbon allotrope, C_5_, predicted by Wei et al. [33] using the CALYPSO code. Note that the initial *tet*-C_4_ has a **dia** topology.

**Figure 2:**
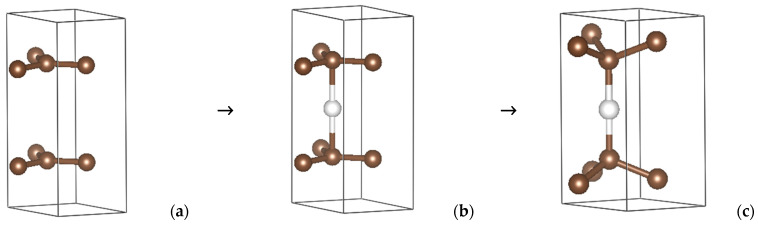
Transformation (and stoichiometry change) from layered *h*-C_4_ (**a**) to C_5_ (**b**) by inserting a carbon atom at z = ½ (white sphere), and the fully geometry-optimized 3D *h*-C_5_ (**c**) (see Table 1 and text).

***h*-C_5_**. Lonsdaleite (space group P6_3_/*mmc*, No. 194) is the rare hexagonal form of diamond, *h*-C_4_, which is characterized by a single four-fold atomic position for carbon at ⅓, ⅔, and z with a small (0.06275) z-value along the hexagonal vertical axis. Changing z to 0 gives a 2D graphite-like carbon structure (Figure 2a) with **hcb** topology. Then, the puckering of the graphite-like layers is a key factor in the transition from a 2D to 3D structure. Here, we modeled the 2D → 3D transformation by inserting a carbon atom between the carbon layers, as shown by the white sphere in Figure 2b. Subsequent geometry relaxation yielded a new 3D structure with C_5_ stoichiometry (Figure 2c). The crystal data with three different carbon positions and a symmetry reduction to *P*-6*m*2 (No. 187) are presented in Table 1. The topology is now **lon**, i.e., lonsdaleite-like. The bottom row shows the total energy after full geometry relaxation, which is found to be larger for *tet*-C_5_ than for *h*-C_5_. The result can be translated into the average cohesive energy per atom, E_coh_/atom, which is obtained after subtracting the atomic energy of a carbon atom in a large box, i.e., −6.6 eV. Then, the cohesive energy is −2.05 eV for *tet*-C_5_ versus −1.90 eV for *h*-C_5_. Both values remain smaller than E_coh_/atom = −2.49 eV for diamond.

### 3.2. New Tetragonal and Hexagonal Boron Nitrides

**Figure 3:**
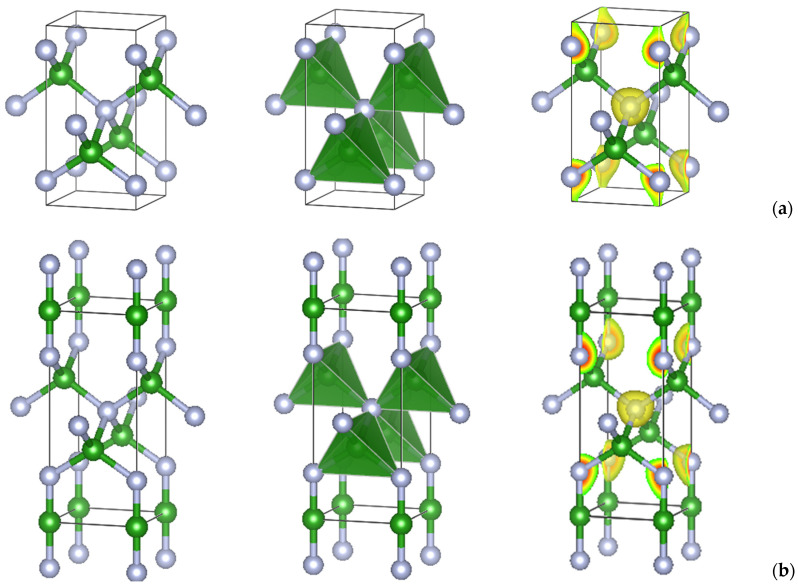
Ball-and-stick (**left**), polyhedral (**middle**), and charge projection (**right**) representations of the crystal structures of new tetragonal B_2_N_3_ (**a**) and B_3_N_3_ (**b**). Green and gray spheres represent boron and nitrogen atoms, respectively.

**Figure 4:**
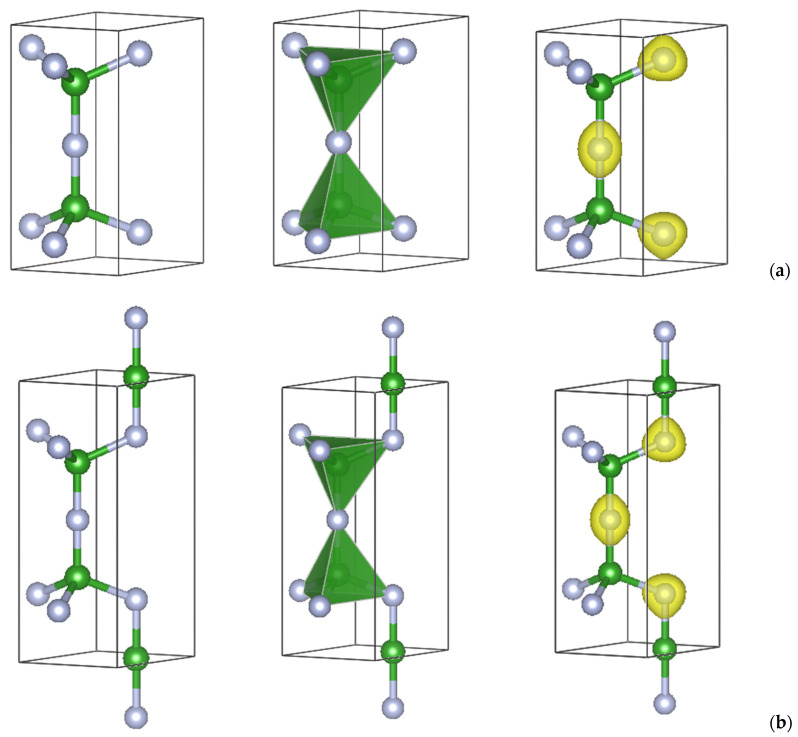
Ball-and-stick (**left**), polyhedral (**middle**), and charge projection (**right**) representations of the crystal structures of new hexagonal B_2_N_3_ (**a**) and B_3_N_3_ (**b**). Green and gray spheres represent boron and nitrogen atoms, respectively.

**B_2_N_3_**. The C_5_ structure was subsequently used as a template to design boron sesquinitride, B_2_N_3_, in both the tetragonal and hexagonal forms. Table 2 shows the corresponding atomic substitutions of carbon with boron and nitrogen at the three different positions in Table 1. The geometry-converged atomic positions were found to be close to the matrix C_5_ allotropes. It can be observed that the cell volumes of *tet*-B_2_N_3_ and *h*-B_2_N_3_ are almost the same as well as the total energies with, however, a slightly lower value for the former. The crystal structures are shown in Figure 3 and Figure 4 in ball-and-stick and tetrahedral representations.

**B_3_N_3_**. Subsequently, equiatomic boron nitride, B_3_N_3_, was designed in both crystal systems by inserting an extra boron at 0, 0, 0 for *tet*-B_3_N_3_ and at ⅓, ⅔, 0 for *h*-B_3_N_3_, as shown in Table 2 (the second and fourth data columns). The resulting structures after full geometry relaxation are shown in Figure 3 and Figure 4. They exhibit preserved, pristine boron sesquinitride structures expanded with additional boron, thus achieving equiatomic stoichiometry. The total energies favor *tet*-B_3_N_3_, which also has a larger volume, indicating a lower density.

Table 2 shows the respective topologies of the new boron nitrides: **3,4^2T1-CA** for the tetragonal phases (like pristine C_5_), **lon** for *h*-B_2_N_3_, and **tfi** for *h*-B_3_N_3_.

## 4. Projections of the Charge Densities

To illustrate the electron distribution within the new boron nitrides, the analysis was extended to a qualitative representation of the charge densities. Figure 3 and Figure 4 (right panels) represent the charge density projections with yellow volumes around the atoms. In all cases, the charges are concentrated around nitrogen atoms (gray spheres). In fact, boron nitride is a polar covalent compound with a significant charge transfer to N, which is characterized by an electronegativity of χN = 3.04 and χB = 2.04 according to the Pauling scale. In B_2_N_3_, the charge gray volumes are squeezed toward the next cell along the vertical *c* direction, corresponding to the N–N connections between successive cells. By introducing new B–N bonds, equiatomic *tet*-B_3_N_3_ and *h*-B_3_N_3_ are created, and the charge volumes are around the N atoms in both symmetries. Regarding the iso-surface values, the following trends were observed: B_2_N_3_: 0.325 and B_3_N_3_: 0.293, i.e., a higher value for a higher nitrogen content.

## 5. Mechanical Properties

The analysis of the mechanical properties was carried out by calculating the elastic tensor through finite distortions of the lattice. The calculated sets of elastic constants C_ij_ (i and j correspond to directions) of the four new boron nitrides are given in Table 3. All C_ij_ values are positive, indicating mechanically stable phases. For comparison, the elastic constants of template *tet*-C_5_ and *h*-C_5_ are also given. As expected, the carbon allotropes have the largest C_ij_ values compared to those of the compounds of the B–N system. Elastic tensor analysis was performed to obtain the bulk (*B*_v_), shear (*G*_v_), and Young’s (*E*_v_) moduli and Poisson’s ratio (ν) by Voight’s averaging [22] using ELATE software [21]. The calculated elastic moduli, whose values follow the trends observed for C_ij_, are shown in Table 4, along with the Vickers hardness values calculated using four contemporary models of hardness [23,24,25,27] and fracture toughness evaluated using the Mazhnik–Oganov model [23].

Since the thermodynamic model is the most reliable in the case of superhard boron compounds [3,38,39] and shows perfect agreement with the available experimental data for cubic boron nitride [40,41], it is obvious that the hardness values calculated within the empirical Mazhnik–Oganov [23] and Chen–Niu [24] models are not reliable. As for the Oganov–Lyakhov model [27], it gives slightly underestimated values, as has already been observed for the superhard compounds of the B–C–N system [38]. It is evident (see Table 4) that the hardness of B_2_N_3_ (52 GPa for both tetragonal and hexagonal polymorphs) is only slightly lower than that of cubic (55 GPa) and wurtzite (54 GPa) BN polymorphs. In contrast, the hardness of B_3_N_3_ is significantly lower, especially for the tetragonal polymorph (42 GPa). However, all four new boron nitrides have a Vickers hardness exceeding 40 GPa, making them members of the superhard phase family.

The fracture toughness of new boron nitrides decreases from 5.5 MPa·m^½^ for *h*-B_2_N_3_ to 3.8 MPa·m^½^ for *tet*-B_3_N_3_, exceeding that of cubic BN (K_Ic_ = 2.8 MPa·m^½^ [39]).

## 6. Energy–Volume Equations of State

**Figure 5:**
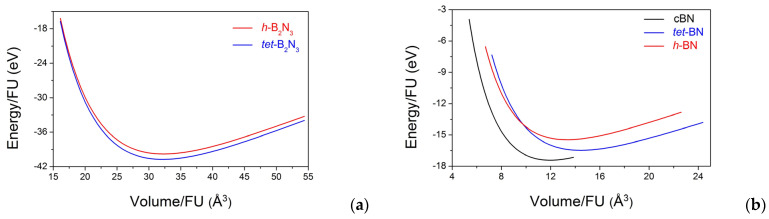
Calculated total energy as a function of volume for new boron nitrides: B_2_N_3_ (**a**) and B_3_N_3_ (**b**). In the case of B_3_N_3_, all values are given per BN formula unit for comparison with cBN.

To determine energy trends when considering the different crystal structures of a solid, it is necessary to establish the corresponding equations of state (EOS). It is important to note that one cannot rely on the quantities obtained from lattice optimizations alone, especially when comparing the energies and volumes of the different phases. The underlying physics means that the calculated total energy corresponds to the cohesion within the crystal, and the solutions to the Kohn–Sham DFT equation give the energy in terms of infinitely separated electrons and nuclei. The zero of the energy depends on the choice of the atomic potentials (projector augmented waves (PAWs) as, here, ultra-soft pseudo-potentials (US-PP), etc.); then, it becomes arbitrary by its shift, not by scaling. However, the energy derivatives and the EOS remain unchanged. Therefore, it is necessary to obtain the EOS and extract the fit parameters to evaluate the equilibrium values. This was conducted via a series of calculations of the total energy as a function of volume for the tetragonal and hexagonal phases of new B–N compounds. The resulting *E*(V) curves, shown in Figure 5, were fitted to the third-order Birch equations of state [42]:

*E*(V) = *E*_0_(V_0_) + (9/8)∙V_0_*B*_0_[([(V_0_)/V])^⅔^ − 1]^2^ + (9/16)∙*B*_0_∙(*B*′ − 4)∙V_0_[([(V_0_)/V])^⅔^ − 1]^3^,

where *E*_0_, V_0_, *B*_0_, and *B*′ are the equilibrium energy; volume; bulk modulus; and its first pressure derivative, respectively. The calculated values are summarized in Table 5. In the case of boron sesquinitride B_2_N_3_ (Figure 5a), the E(V) curve of *tet*-B_2_N_3_ remains at a slightly lower energy and a higher volume than *h*-B_2_N_3_, but both curves remain close. Quantitatively, this is translated by close equilibrium values (Table 5) due to the close densities of the two phases. Larger differences are observed for the equiatomic phases (Figure 5b), where the tetragonal B_3_N_3_ systematically has lower energy and a larger volume than the hexagonal one. An intersection of the *E*(V) curves is observed at a volume of 9.74 Å^3^ per BN formula unit. The corresponding pressure of 200(30) GPa was estimated by the Murnaghan equation [43] using the V_0_, *B*_0_, and *B*′ values from Table 5.

As can be seen in Figure 5b, both B_3_N_3_ polymorphs are metastable with respect to cubic BN over the whole range of experimentally accessible pressures. Nevertheless, the closeness of their cohesive energies allows for the possibility of the formation of both B_3_N_3_ polymorphs at high pressures and high temperatures as a result of alternative metastable behavior.

## 7. Dynamic and Thermodynamic Properties

### 7.1. Phonons Band Structures

**Figure 6:**
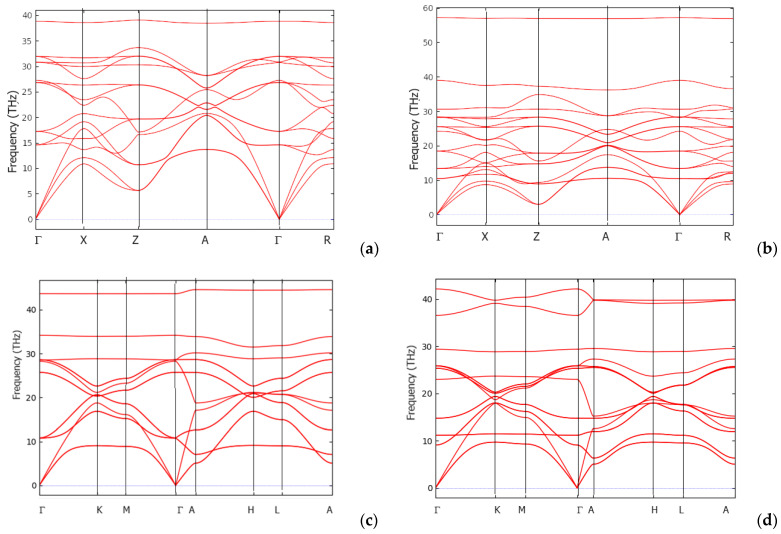
Phonon band structures of new boron nitrides along the major directions of the simple tetragonal (**a**,**b**) and hexagonal (**c**,**d**) Brillouin zones: *tet*-B_2_N_3_ (**a**); *tet*-B_3_N_3_ (**b**); *h*-B_2_N_3_ (**c**); *h*-B_3_N_3_ (**d**).

To verify the dynamic stability of the new B–N phases, an analysis of their phonon properties was performed. The phonon band structures obtained from the high resolution of the tetragonal and hexagonal Brillouin zones in accordance with the method proposed by Togo et al. [28] are presented in Figure 6. The bands (red lines) develop along the main directions of the tetragonal (or hexagonal) Brillouin zone (horizontal *x*-axis) and are separated by vertical lines for enhanced visualization, while the vertical direction (*y*-axis) represents the frequencies ω, given in terahertz (THz).

The band structures include 3N bands: three acoustic modes starting from zero energy (ω = 0) at the Γ point (the center of the Brillouin zone) and reaching up to a few terahertz and 3N-3 optical modes at higher energies. The low-frequency acoustic modes are associated with the rigid translation modes (two transverse and one longitudinal) of the crystal lattice. The calculated phonon frequencies are all positive, indicating that the four new B–N phases are dynamically stable.

In the case of the tetragonal phases, in addition to the dispersed bands, the flat bands at ~39 THz for B_3_N_2_ (Figure 6a) and the higher frequency band at ~58 THz for B_3_N_3_ (Figure 6b) are observed. The 39 THz band for tetragonal B_2_N_3_ can be attributed to the B–N distance within the tetrahedron, while the 58 THz band for tetragonal B_3_N_3_ can be assigned to the B–N distance in the B-N-B fragment along the *c*-axis. For hexagonal phases, such flat bands are absent, while observed bands with frequencies of about 40 THz are due to the larger B–N distances in these phases (see Table 2).

### 7.2. Temperature Dependence of the Heat Capacity

**Figure 7:**
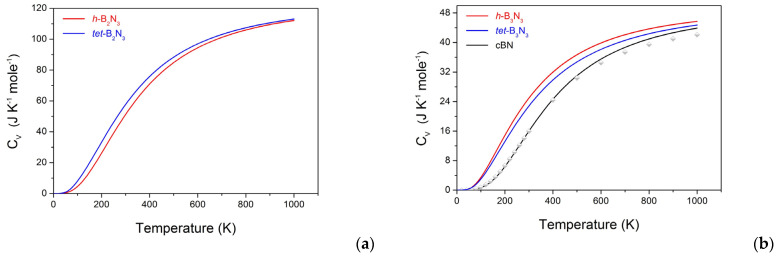
Heat capacity at constant volume (C_V_) of new boron nitrides: B_2_N_3_ (**a**) and B_3_N_3_ (**b**). In the case of B_3_N_3_, C_V_ values are given per BN formula unit for comparison with cBN. Experimental heat capacity data for cBN [45] are shown as gray symbols.

The thermodynamic properties of the new B–N phases were calculated from the phonon frequencies using the statistical thermodynamic approach [44] on a high-precision sampling mesh in the Brillouin zone. The temperature dependencies of the heat capacity at constant volume (C_V_) for all new boron nitrides are presented in Figure 7 and for B_3_N_3_ in comparison with the available experimental C_p_ data for cubic BN [45]. The heat capacity of tetragonal B_2_N_3_ is slightly higher than that of the hexagonal phase (Figure 7a), while the opposite is observed for B_3_N_3_ (Figure 7b). The heat capacities of both B_3_N_3_ polymorphs are slightly higher than that of cubic BN, which is consistent with their more open structures compared to the dense cubic structure. The observed excellent agreement between the calculated and experimental data for cBN supports the validity of the method used to estimate the thermodynamic properties of new boron nitrides.

## 8. Electronic Band Structures

**Figure 8:**
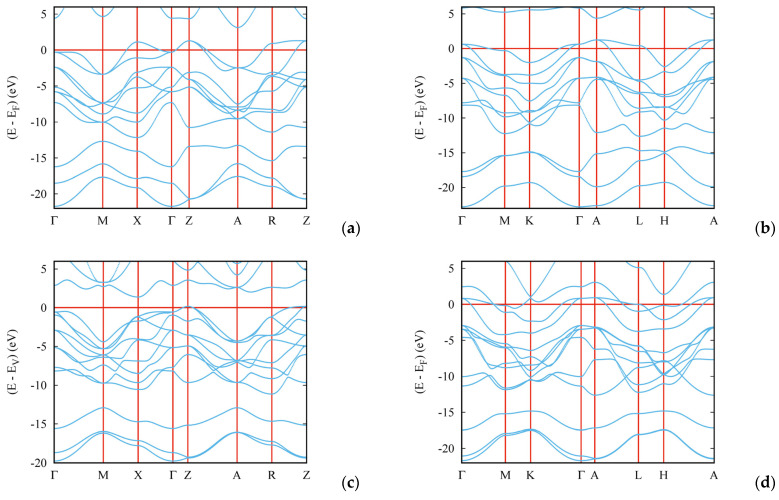
Electronic band structures of new boron nitrides: *tet*-B_2_N_3_ (**a**); *h*-B_2_N_3_ (**b**); *tet*-B_3_N_3_ (**c**); *h*-B_3_N_3_ (**d**).

The electronic band structures of the new boron nitrides were calculated using the all-electron DFT-based augmented spherical wave (ASW) method [29] using the crystal structure data from Table 1. The results are shown in Figure 8. The bands (blue lines) develop along the main directions of the respective tetragonal and hexagonal Brillouin zones. The zero energy along the vertical axis is considered with respect to the Fermi level E_F_. For both B_2_N_3_ polymorphs (Figure 8a,b), the bands cross the Fermi level with a tendency toward a weakly metallic behavior. Obviously, a semiconducting behavior is observed for *tet*-B_3_N_3_, where a small gap develops (Figure 8c); hence, the energy reference is now at E_V_, i.e., at the top of the valence band. Finally, *h*-B_3_N_3_ is clearly metallic with several bands crossing E_F_ (Figure 8d). The different behaviors observed between the two equiatomic phases can be attributed to the fact that *tet*-B_3_N_3_ has considerably lower energy than *h*-B_3_N_3_, as shown in Table 2. As a result, its electronic structure is closer to that of cubic BN. Thus, the new B–N phases exhibit different electronic properties.

## 9. Conclusions

The present work involved a challenging prediction of new tetragonal and hexagonal boron nitrides, B_2_N_3_ and B_3_N_3_, from crystal chemistry and first principles using original pentacarbon templates. The crystal chemistry investigations were supported by computations of the ground structures and energy-dependent quantities within the well-established framework of quantum density functional theory (DFT). All new phases were found to be cohesive. The mechanical stability of the new phases, which follows from the calculated values of the elastic constants, is coupled with their extreme hardness, varying from 45 GPa for *tet*-B_3_N_3_ to 52 GPa for both B_2_N_3_ polymorphs. Dynamically, all new phases were found to be stable from positive phonon frequencies, and observed high-frequency modes were assigned to the short B–N distances in their crystal structures. Conductive electronic behavior is observed, which varies from small bandgap semiconducting *h*-B_2_N_3_, to weakly metallic tetragonal B_2_N_3_ and B_3_N_3_, and finally to metallic *h*-B_3_N_3_. The results obtained are expected to inspire experimental attempts to synthesize new B–N phases at high pressures and high temperatures.

## Figures and Tables

**Table 1:** Crystal structure transformations of tetragonal carbon (from C_4_ to C_5_) and from 2D-C_4_ to 3D-C_5_ (hexagonal setups). See text for details.

Space Group Topology	*tet*-C_4_ *I*-4*m*2 (No. 119) dia	*tet*-C_5_ *P*-4*m*2 (No. 115) 3,4^2T1-CA	2D C_4_ *P*6*mm* (No. 184) hcb	3D C_5_ *P*-6*m*2 (No. 187) lon
*a*, Å	2.527	2.480	2.461	2.487
*c*, Å	3.574	4.990	6.698	5.581
Atomic positions	C1a (½, ½, ½) **→** C1b (0, 0, 0) **→** C2a (½, 0, ¼) **⌉ →** C2b (0, ½, ¾) **⌋**	C1 (1c) (½, ½, ½) C2 (2e) (0, 0, ±z) z = 0.313 C3 (2g) (0, ½, ±z′) z′ = 0.854	C(4b) (⅓, ⅔, 0)	C1 (2h) (⅓, ⅔, z) z = 0.861 C2 (2i) (⅔, ⅓, z′) z′ = 0.762 C′(2f) (⅔, ⅓, ½)
E_total_, eV E_coh_/atom, eV	−36.38 −2.49	−43.26 −2.05	−36.87 −2.67	−42.51 −1.9

N.B. E(C) = −6.6 eV. E_coh_/atom (diamond) = −2.49 eV.

**Table 2:** Crystal structure parameters of new B–N phases.

Space Group Topology	*tet*-B_2_N_3_ *P*-4*m*2 (No. 115) 3,4^2T1-CA	*tet*-B_3_N_3_ *P*-4*m*2 (No. 115) 3,4^2T1-CA	*h*-B_2_N_3_ *P*-6*m*2 (No. 187) lon	*h*-B_3_N_3_ *P*-6*m*2 (No. 187) tfi
*a*, Å	2.523	2.636	2.564	2.541
*c*, Å	4.944	6.110	5.504	7.020
V_cell_, Å^3^	31.47	42.45	31.33	39.25
Density, g/cm^3^	3.358	2.913	3.374	3.150
Shortest B–N bond, Å	1.35	1.32	1.41	1.44
Atomic positions	B1(2g) (0, ½, 0.310) N1 (2e) (0, 0, 0.863) N2 (1c) (½, ½, ½)	B1(2g) (0, ½, 0.353) B2(1a) (0, 0, 0) N1(2e) (0, 0, 0.783) N2(1c) (½, ½, ½)	B (2i) (⅔, ⅓, 0.757) N1(2h) (⅓, ⅔, 0.865) N2(1f) (⅔, ⅓, ½)	B1 (2i) (⅔, ⅓, 0.704) B2 (1c) (⅓, ⅔, 0) N1(2h) (⅓, ⅔, 0.209) N2(1f) (⅔, ⅓, ½)
E_total_, eV	−40.76	−49.43	−39.78	−46.42

**Table 3:** Elastic constants (C_ij_) of the new boron nitrides in comparison with those of the original carbon allotropes (all values are in GPa).

	C_11_	C_12_	C_13_	C_33_	C_44_	C_66_
*tet*-C_5_	943	9	136	1194	198	337
*tet*-B_2_N_3_	712	57	164	987	82	307
*tet*-B_3_N_3_	614	23	126	928	119	161
*h*-C_5_	920	95	46	1453	412	333
*h*-B_2_N_3_	680	137	20	1416	272	199
*h*-B_3_N_3_	453	124	139	1190	165	154

**Table 4:** Mechanical properties of new boron nitrides: Vickers hardness (*H*_V_), bulk modulus (*B*), shear modulus (*G*), Young’s modulus (*E*), Poisson’s ratio (ν), and fracture toughness (*K*_Ic_). The subscript _V_ for elastic moduli indicates the use of the Voigt averaging scheme. # denotes the space group number. The corresponding values for wurtzite and cubic boron nitrides are given for comparison.

	*H*_V_				*B*		*G*_V_	*E*_V_	*ν*_V_	*K*_Ic_^‡^
	T *	LO ^†^	MO ^‡^	CN ^§^	*B*_0_ *	*B*_V_				
	GPa									MPa·m^½^
*tet*-B_2_N_3_ ^#115^	52	51	23	26	357	353	229	566	0.233	4.5
*h*-B_2_N_3_ ^#187^	52	49	49	51	359	348	322	738	0.146	5.5
*tet*-B_3_N_3_ ^#115^	45	42	21	26	314	301	205	502	0.222	3.8
*h*-B_3_N_3_ ^#187^	49	46	22	25	340	322	210	517	0.233	3.9
*w*-BN ^#186^	54	50	70	64	375 [34]		384 [35]	858 **	0.118 **	–
*c*-BN ^#216^	55	50	74	69	381 [36]		399 [36]	890 **	0.107 **	2.8 [37]

* Thermodynamic model [26]; ^†^ Lyakhov–Oganov model [27]; ^‡^ Mazhnik–Oganov model [23]; ^§^ Chen–Niu model [24]; ** calculated using isotropic approximation.

**Table 5:** Calculated properties of new boron nitrides: bulk modulus (*B*_0_) and its first pressure derivative (*B*_0_′); total energy (*E*_0_) and equilibrium volume (V_0_) per formula unit (FU).

	B_2_N_3_		B_3_N_3_	
	Tetragonal	Hexagonal	Tetragonal	Hexagonal
*B*_0_ (GPa)	344	336	300	317
*B*_0_′	3.76	3.75	3.67	3.71
*E*_0_/FU (eV)	−40.7	−39.1	−49.4	−46.4
V_0_/FU (Å^3^)	31.5	42.5	31.3	39.3

## Data Availability

The data presented in this study are available on reasonable request.
